# New insight into teratogenic effects of (*S*)-thalidomide in zebrafish embryos growing inside the chorion and subjected to electric pulses

**DOI:** 10.1038/s41598-025-00641-y

**Published:** 2025-05-16

**Authors:** Anna Małkowska, Łukasz Szymański, Julita Nowakowska, Dorota Kaczorek, Agnieszka Nosal-Wiercińska, Robert Kawęcki, Ireneusz P. Grudzinski, Anna M. Nowicka

**Affiliations:** 1 Department of Toxicology and Food Science, Faculty of Pharmacy, Medical University of Warsaw, Banacha 1 Str., Warsaw, 02-097 Poland; 2 University of Warsaw, Faculty of Chemistry, Pasteura 1 Str., Warsaw, 02-093 Poland; 3 University of Warsaw, Faculty of Biology, Miecznikowa 1 Str., Warsaw, 02-096 Poland; 4 University of Siedlce, Faculty of Natural Science, 3 Maja 54 Str., Siedlce, 08-110 Poland; 5 Maria Curie-Sklodowska University in Lubin, Institute of Chemical Sciences, Departament of Analytical Chemistry, Maria Curie-Sklodowska 3 Str., Lublin, 20-031 Poland

**Keywords:** Thalidomide, Electroporation, Zebrafish embryos, Teratogenicity, Biophysics, Drug safety, Toxicology

## Abstract

Studies of (*S*)-thalidomide were conducted on zebrafish embryos subjected to electroporation processes using a square wave pulse generator. The results showed that the electroporation increases the absorption of (*S*)-thalidomide through the chorion into the growing embryos, which was confirmed by increased thalidomide levels in the embryo bodies at different hours post-treatments using differential pulse voltammetry and controlled-growth mercury drop electrode techniques. (*S*)-thalidomide administered by electroporation produced structural body deformations in zebrafish embryos as showed by scanning electron microscopy studies. Detailed transmission electron microscopy analysis evidenced multiple deposits of the outer layer and translucencies in the chorion structure, which was also pronounced on the mitochondrial membranes. The results confirmed the spontaneous conversion of the (*S*)-thalidomide enantiomer to the (*R*)-enantiomer in embryos exposed to the (*S*)-thalidomide only and subjected to electroporation pulses. The electroporation was found as a promising method to increase the uptake of (*S*)-thalidomide in the developmental studies on early zebrafish embryos growing in the chorion.

## Introduction

In 1954, thalidomide was synthesized and introduced into the market in Germany as a safe nonbarbiturate over-the-counter sedative but also for the treatment of nausea and vomiting associated with morning sickness during pregnancy. At that time, thalidomide was not approved by the U.S. FDA and was withdrawn from the market in 1961 in the wake of a series of devastating congenital disabilities in newborn babies. These anomalies involved mainly the limbs (particularly upper limbs), face, eyes, ears, genitalia, and internal organs, including the heart, kidneys, and gastrointestinal tract. Furthermore, the vertebral column was also affected in some cases, and the occurrence of facial palsies was also documented^1^. Importantly, before thalidomide was first introduced into the market, no teratogenic effects had been observed on mice or rats^2^. It was not until later that thalidomide was discovered teratogenic effect in rabbits and chicks^3^. Therefore, the thalidomide tragedy prompted researchers all over the world to conduct toxicological studies on different animal species^4,5^. One of the widely used vertebrate model organism in toxicological studies in zebrafish (Danio rerio)^6,7^. This model has gained popularity due to genetic similarities between zebrafish and humans, which translate to similar pharmacologic effects^8^ and systemic toxicities, including teratogenic effects, in those species^9–11^. The model is cost-effective for high-throughput screening assays in toxicology due to the species’ high fecundity, rapid embryonic development, and fast and transparent organogenesis^8^. In recent years, thalidomide has, once again, attracted clinical interest due to the discovery of its therapeutic effects in patients with multiple myeloma and erythema nodosum leprosum^12^. Moreover, it can be helpful in the treatment of various disorders and diseases including cancer^13^, Crohn’s disease, Behcets disease, systemic lupus erythematosus, HIV infections, and other conditions^1^. There have also been reports on the use of thalidomide in diabetic retinopathy^14^ and in the treatment of chronic pain^15^. Despite the many years that have passed since the thalidomide tragedy, it is still the subject of many scientific papers due to its renaissance in clinical practices. Observing the additional effects of thalidomide and new medical indications required a thorough understanding of the mechanism of action and the biotransformation of thalidomide in well-predictive species such as zebrafish embryos. It was reported that zebrafish were sensitive to thalidomide, observing defects of pectoral fins and other deformities like a vascular defect, defect of the dorsal artery, shortness in length, loss of blood flow, severe pericardial edema, and heart failure or otic vesicle^11,16^. Thalidomide alters molecules crucial to vascular development, including vascular endothelial growth factor, a key signaling molecule in vessel growth and maturation^17^ and failure of angiogenic growth is the cause of thalidomide-induced pectoral fin malformations in zebrafish^18^. It is well-known that the teratogenic effect of thalidomide is due to its binding to the cereblon (CRBN) and the inhibition of its associated ubiquitin ligase activity^19^. Note that the CRBN is now considered a primary direct target for both the anti-cancer activity and teratogenicity of thalidomide^19^. Those findings confirmed the presumed impact of thalidomide on the development and shortening of pectoral fins in zebrafish embryos^11^. The lack of fins in zebrafish following exposure to thalidomide was observed in experiments involving dechorionated embryos^16,20^ or after microinjection^21^. This deformity was absent in embryos without dechorionation^9^.

Studies evidence that thalidomide affects blood vessel remodeling in Fli-EGFP transgenic zebrafish and is responsible for discontinuous development and blood vessel occlusion of the dorsal longitudinal anastomotic vessel and intersegmental blood vessel^22^. Mori et al. have noticed differences between the two enantiomers and also confirmed this finding in the zebrafish model. Thalidomide is a stereo-isomer and can exist in two enantiomeric forms: (*S*) and (*R*), depending on the configuration of chemical groups bound to the chiral carbon. Each configuration results in a different structure and properties. The (*S*)-enantiomer of thalidomide is believed to be teratogenic and toxic, while the (*R*)-enantiomer has a neutral, sedative effect. However, it should be noted that thalidomide in the body can freely change from one form to another, therefore, a level of exposure determined to have no toxic effects on humans or animals called as a “safe” dose may be subject to error^23^. The (*S*)-enantiomer of thalidomide induced more pronounced teratogenic effects on fin development in zebrafish than the (*R*)-enantiomer. Thalidomide (*S*)-enantiomer is more effective at inhibiting TNF-α production and angiogenesis compared to the (*R*)-enantiomer^16^. The authors explain the 10-fold stronger binding affinity of CRBN to the (*S*)-enantiomer than that to the (*R*)-enantiomer^16^. In 2023 Dong et al. noticed pectoral fin defects and other malformations including pericardial edema, and defects of otic vesicles including otoliths in hCYP3A7 zebrafish but not in wild-type zebrafish. They also confirmed a reduction in fgf8 expression in pectoral fin buds only in hCYP3A7-expressing embryos/larvae and suggested the contribution of human CYP3A in the teratogenicity of thalidomide^24^.

Most of the published work has been done on thalidomide racemates containing both enantiomers and dosed into the dechorionated zebrafish embryos or directly injected into the embryos. In our study, we aim to investigate the effect of (*S*)-thalidomide due to its well-known teratogenic effects and to assess the possibility of its conversion to the *(R*)-enantiomer in the early zebrafish embryos. Due to the potential limitations in the passage of (*S*)-thalidomide through the chorion, the zebrafish embryos have been subjected to electroporation processes using a square wave pulse generator. The morphological changes following exposure of embryos to (*S*)-thalidomide with and without electroporation were studied. Moreover, the content of the thalidomide isomers was determined in the zebrafish embryos undergoing the electroporation and compared with those in the embryos exposed to (*S*)-thalidomide but not electroporated.

## Experimental setup

### Materials

All used chemicals: (*S*)-thalidomide ((*S*)-TD), dimethyl sulfoxide (DMSO), dichloromethane (DCM), tetrabutylammonium hexafluorophosphate (Bu_4_NPF_6_), L–methionine (MT), sodium perchlorate (NaClO_4_), and perchloric acid (HClO_4_) were of the highest available quality and were purchased from Sigma Aldrich. The Millipore DirectQ UV3 system was used as the source of water (*R* > 18 MΩ·cm).

### Zebrafish experiments

Zebrafish embryos (AB×TL) were obtained from the International Institute of Molecular and Cell Biology in Warsaw and maintained in E3 medium. The embryos were identified according to Kimmel et al.^25^, and only the fertilized ones that showed the process of cell division were selected. At 3 h post fertilization (hpf), the embryos were placed on 96-well plates (one embryo per well, twenty for one group) with previously.

(*S*)-thalidomide in a concentration 0.8 mM. Observations were made at 24-hour intervals up to 96 hpf. Currently, the European Commission Directive 2010/63/EU, permits experimentation in fish embryos at earliest life stages without being regulated as animal experiments; zebrafish are considered models in vitro until 120 hpf (http://data.europa.eu/eli/dir/2010/63/2019-06-26; accessed 9 December 2021 EFSA opinion: 10.2903/j.efsa.2005.292; accessed 9 December 2021). The plates were incubated at a constant temperature of 27 ± 1 °C with a light-dark cycle (12 h/12 h) throughout the study period. The embryos were analyzed under a microscope (Olympus CKX53), and images were captured using an Olympus EP50 camera (CAM-EP50) The length and width of embryos after hatching were measured using EPview1.3 software at 96 hpf. A tricaine (0.3%) solution was applied at the end of the experiment for euthanasia.

### Electroporation process

An electroporation method was applied using the ECM 830 BTX system (a square wave pulse generator used in both in vitro and in vivo studies) to facilitate the introduction of thalidomide into zebrafish embryos. Our study involved meticulously developing electric parameters, including voltages (10, 20, 25, and 30 eV), pulse duration (10 or 20 ns), and several pulses (3×, 6×, 12×). This optimization aimed to enhance chorion permeability in zebrafish embryos without causing damage, a crucial step in our research. The time of electroporation is also significant, and with the same conditions, the survival of the embryos is much more versatile when electroporation is conducted at 1 hpf. We noticed a 50% decreased survival compared to embryos exposed after 4 h. Already within 24 h, many embryos coagulated were treated with electric pulses in the early stages of a lifetime (1 hpf). Considering the above information, we decided to subject embryos at 4 hpf to electroporation. In the next step, the fertilized zebrafish embryos were treated with electroimpulses in the presence and the absence of (*S*)-thalidomide under strictly defined conditions, which we had established as safe for embryos.

### Differential pulse voltammetry

Differential pulse voltammetry (DPV) was performed in the three-electrode system using an Autolab Eco Chemie potentiostat, model PGSTAT 12. The disc glassy carbon electrode (GC, *φ* = 3 mm, BAS Instruments) was used as a working electrode, an Ag/AgCl/3 M KCl as a reference electrode, and a platinum wire as the auxiliary electrode. During all measurements, to minimize the electrical noise the electrochemical cell was kept in a Faraday cage. The measurements were carried out with deoxygenated solutions. The voltammetric analysis of the content of both thalidomide enantiomers in solution (in which embryos were stored) and zebrafish embryos homogenates exposed to 0.8 mM (*S*)-TD (the samples were homogenized with 200 µL 0.1% DMSO for 1 min.) at different stages of their development without and with electroporation was performed using the earlier developed by us sensor^26^. The recognition process of the thalidomide enantiomers was based on the unique specific interaction between (*S*)-thalidomide and (*R*,*R*)-NDI derivative and (*R*)-thalidomide and (*S*,*S*)-NDI. According to our earlier studies the formed complexes (*S*)-TD—(*R*,*R*)-NDI and (*R*)-TD—(*S*,*S*)-NDI is stabilized by two hydrogen bonds between the (*R*)-NDI-1 sulphonamide moiety and glutarimide ring in TD. Moreover the (*S*)-TD can additionally form the *π*-*π* stacking and *π*-lone pair interactions. The sensors used in the study were characterized by a wide range of linear responses from 5·10^−4^ to 10 mg·L^−1^ and low values of the limit of detection: 2.1·10^−5^ and 2.4·10^−5^ mg·L^−1^, respectively for (*S*)-TD sensor and (*R*)-TD sensor.

### Adsorption measurements

The measurements were performed in a three–electrode cell containing: dropping mercury–electrode (CGMDE) with a controlled increase rate and a constant drop surface (0.014740 cm^2^, as a working electrode (MTM Poland); Ag/AgCl (3.0 M saturated solution of NaCl) as a reference electrode and a platinum spiral, as an auxiliary electrode. The solutions were deaerated using nitrogen, which was passed over the solutions during the measurements. The supporting electrolyte was a mixture of 1.0 mol·L^−1^ NaClO_4_ and 1.0 mol·L^−1^ HClO_4_. The concentrations of (*S*)-TD were in the range of 0.1–1.0·10^−3^ mol·L^−1^, while MT was in the range 0.1·10^−3^ – 5.0·10^− 3^ mol·L^−1^. The double layer capacity (*C*_d_) was measured using the AC impedance technique with the Autolab/GPES (Version 4.9) (Eco Chemie). The reproducibility of the average capacity measurements was ± 0.5%. For the whole polarization range, the capacity dispersion was tested at different frequencies between 200 and 1800 Hz. In the studied potential range, dispersion of the capacitance was observed. To obtain the equilibrium values of differential capacity, the linear dependence of capacity on a square element from frequency was extrapolated to zero frequency. This procedure assumes that the impedance of the double-layer is equivalent to a series capacity-resistance combination and the rate of adsorption is diffusion-controlled.

### Transmission electron microscopy (TEM) and scanning electron microscopy (SEM) imaging

For analysis in a TEM, the zebrafish embryos and larvae were fixed in 2.5% glutaraldehyde in 0.1 M cacodylate buffer pH = 7.2 overnight at room temperature. Samples were washed in cacodylate buffer three times and stained with 1.0% osmium tetroxide in bidistillated water for 6 h at room temperature. Afterward, the samples were dehydrated by ethanol solutions of increasing concentration (30% – 10 min, 50% – 10 min, 70% – 24 h, 90% – 10 min, 96% – 10 min, anhydrous EtOH – 10 min, finally acetone – 10 min). After fixation and dehydration, samples were embedded in resin (first mixed with successive acetone solutions of increasing concentration (1:3–30 min, 1:1–2 h, 3:1–5 h) and then pure (for 12 h) and polymerized 24 h at 60 °C in incubator (Agar Scientific). Next, 70 nm sections were cut with a diamond knife on RMC MTXL ultramicrotome (RMC Boeckeler Instruments) to transmission electron microscopy. The sections on grids were contrasted with UranyLess (Micro to Nano) and lead citrate after 2 min. Samples were analyzed in a LIBRA 120 transmission electron microscope (Carl Zeiss), at 120 keV. Photographs were made with a Slow-Scan CCD camera (ProScan), using the EsiVision Pro 3.2 software. Measurements were performed using the analySIS 3.0 image-analytical software (Soft Imaging Systems GmbH). The larvae to be analyzed in SEM after dehydration were dried in a critical point and when dry, they were mounted to an aluminum stub and the samples were sputter coated with a gold alloy. The layers of the alloy were sputtered using a Polaron SC7620 Mini Sputter Coater. Following sputter coating, the samples were viewed with a scanning electron microscope (LEO 1430VP, Zeiss).

### Statistical analysis

The Shapiro-Wilk test was used to assess the normal distribution of the data, while Levene’s test was used to evaluate homogeneity. To identify statistically significant differences between groups, we used either a one-way ANOVA with post-hoc tests. The results were considered significant when the *p* value was ≤ 0.05. Data are presented as mean ± standard deviation (SD). All analyses were conducted using Statistica 13.3 software (StatSoft).

## Results and discussion

### Validation of zebrafish embryos electroporation process

**Fig. 1:**
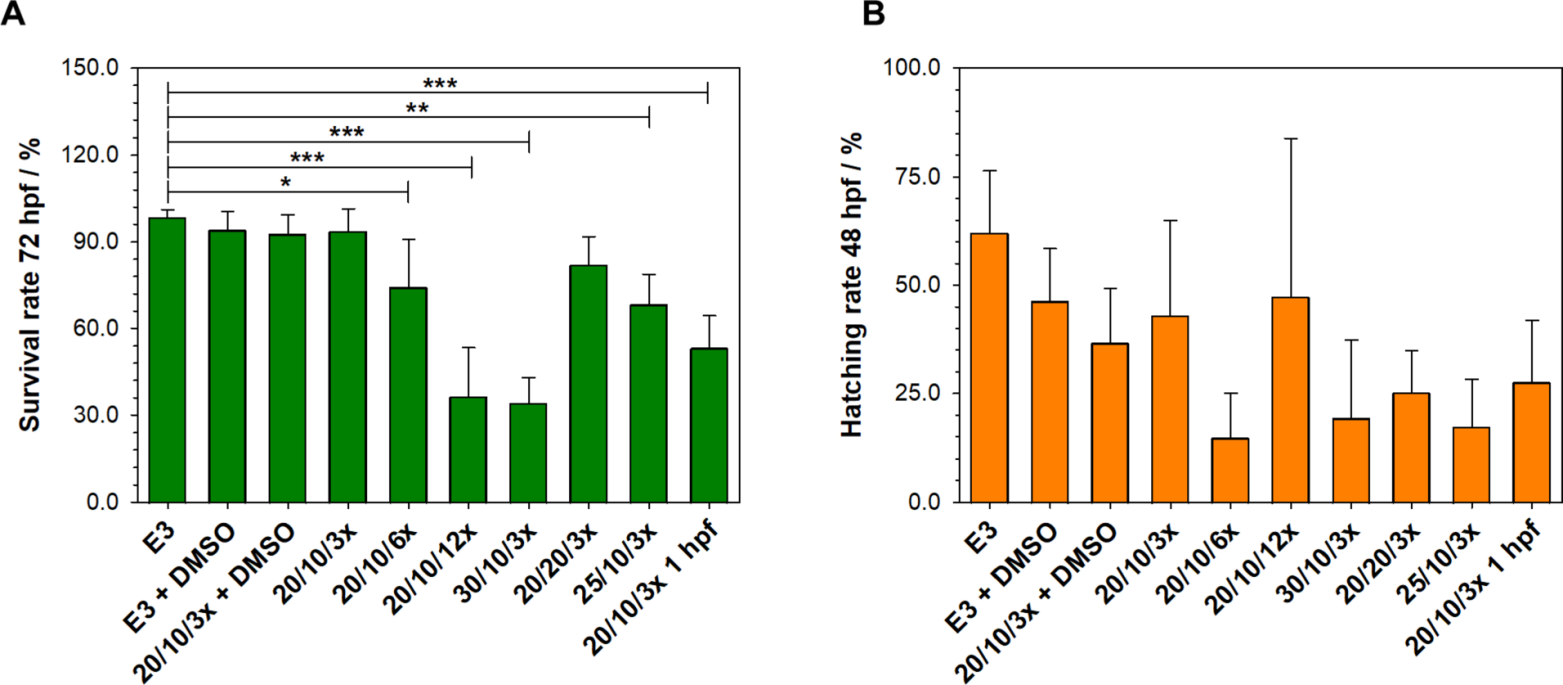
The effects of electroporation in different conditions at 4 hpf (voltage/pulse duration/number of pulses) on zebrafish embryos survival rate (A) and hatching rate (B) compared to the control group exposed to E3 solution or E3 with 0.1% DMSO and in 1 hpf. Voltage/pulse duration/number of pulses were shown on horizontal axes. Values are presented as mean ± standard deviation (SD), *n* = 3 biological replicates with 20 zebrafish embryos per group. one-way ANOVA, followed by Dunnett post hoc test, * indicates a significant difference from controls (*p* < 0.05), **significantly different from controls (*p* < 0.01), ***significantly different from controls (*p* < 0.001).

Validation of zebrafish embryos electroporation process. The embryo is surrounded by a chorion in the first hours after fertilization. The chorion is a semipermeable membrane protecting the embryos from external exposure to xenobiotics. The substance’s impact is the ability to pass through the chorion, which can cause false negative results when examining substances with low permeability through the chorion. The situation is much more complicated when the substances exceed 3 kDa and are not soluble in water^27^. Thalidomide is an example of a compound for which the chorion can act as a barrier to penetration into the embryo. Therefore, in most of the published studies addressing to thalidomide and zebrafish, the chorion was removed before thalidomide treatments^11,16,18^ or thalidomide was directly injected into the embryo yolk^21^. Our experiment used the electroporation method to improve chorion permeability for (*S*)-thalidomide. Electroporation is a process that increases the permeability of the cell membrane by applying an external high-voltage electric field. It is a valuable technology used, among other things, in gene transfection, cancer research, and microbial inactivation^28^. In our experiments, the electroporation process was preliminary validated before starting the exposure with thalidomide. The influence of voltage, pulse duration, and the number of pulses on embryos survival and the speed of chorion exit was arranged. The parameters including voltages (20 eV), pulse duration (10 ns), and a number of pulses (3 pulses) did not affect embryo survival at 72 hpf or delay chorion emergence compared to controls. These parameters were used in experiments with (*S*)-thalidomide. The Fig. 1 shows the effect of electroporation on zebrafish embryos survival rate and hatching rate compared to the control group exposed to E3 solution or E3 with 0.1% DMSO. Exposure conditions at 20 eV with a duration of 10 ms and 3 number of pulses (20/10/3) do not show statistically significant differences compared to controls when applied 4 h after fertilization. The application of the same parameters at 1-hour post-fertilization already causes significant embryo damage during the electroporation process, and the number of embryos that can survive up to 72 h is significantly lower than the control. Electroporation at higher voltages of 25 or 30 eV, or the use of a greater number of pulses even at 20 eV, significantly reduces embryos survival (Fig. 1).

### Morphological changes of *Danio rerio* embryos after (*S*)-thalidomide exposure without and with the electroporation process

**Fig. 2:**
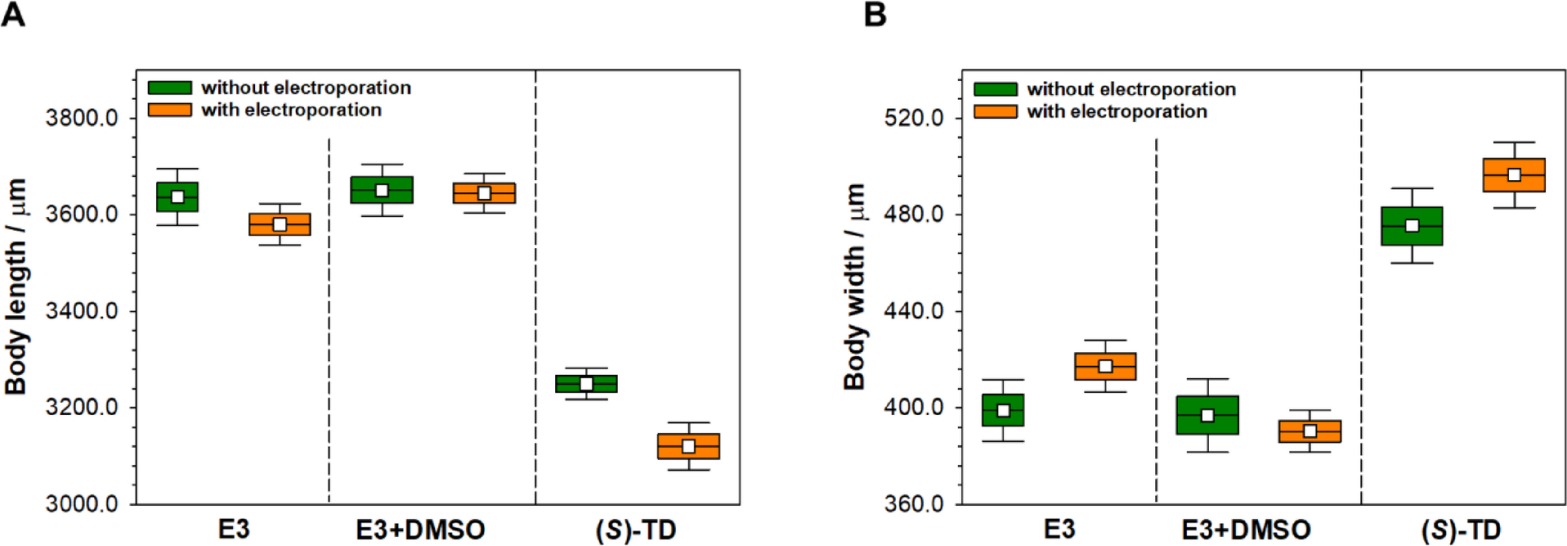
The effect of (*S*)-thalidomide in the concentration of 0.8 mM on the body length (**A**) and the body width (**B**) of zebrafish embryos at 96 hpf. Values are presented as mean ± standard deviation (SD) *n* = 26; one-way ANOVA, followed by Newman-Keuls post-hoc test, * indicates a significantly different (*p* < 0.05), ** significantly different *p* < 0.01.

To more accurately assess the effect of (*S*)-thalidomide, which is considered a teratogenic compound, the zebrafish embryos were electroporated. The studies evidence that (*S*)-Thalidomide alone caused a decrease in the length of embryos and an increase in their width and the use of electroporation intensified this effect (Fig. 2). To data, a statistically significant difference was observed between the length and width of embryos exposed to thalidomide without electroporation and after electroporation. Analyses performed using one-way ANOVA with post hoc test showed electroporation as a significant factor causing reduced embryo length and making them wider in the case of exposure to (*S*)-thalidomide (*p* < 0.01) (Fig. 2). Embryo length measurements are one of the parameters to assess the effect of a substance on the embryonic development of zebrafish^29^. Additionally, embryo length measurement levels eliminate the subjective assessment of the researcher^30^. In the present study, we also measured the embryos width in the yolk region at its widest point in the top view. In typically developing embryos, the yolk slowly disappears, and disturbances in its absorption indicate an adverse effect of the substance under study.

**Fig. 3:**
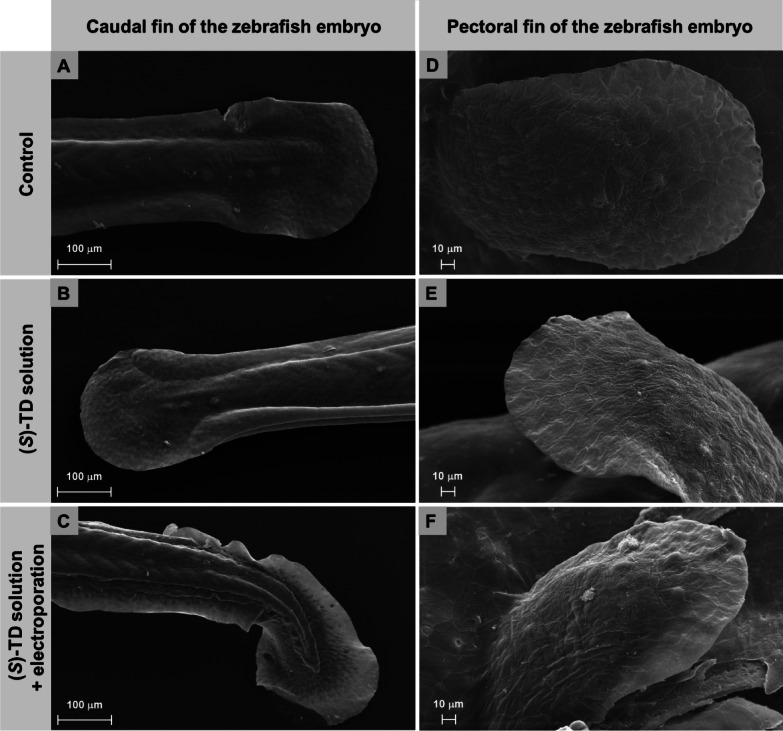
Representative SEM images of the caudal, and pectoral fins of zebrafish embryos at 72 hpf exposed to the (*S*)-thalidomide action and electroporation.

Analysis of the caudal fin structure in the zebrafish embryo treated with (*S*)-thalidomide enantiomer showed a strong deformation in the tail end of the embryo, see Fig. 3A–C. These deformations were visible only in the variant with electroporation applied. On the other hand, analysis of the fin structure showed that the fins in larvae developing in the presence of (*S*)-TD are significantly smaller than those of control larvae and electroporation alone does not affect their development, see Fig. 3D–F.

### Intracellular structural changes induced by the presence of (*S*)-thalidomide

**Fig. 4:**
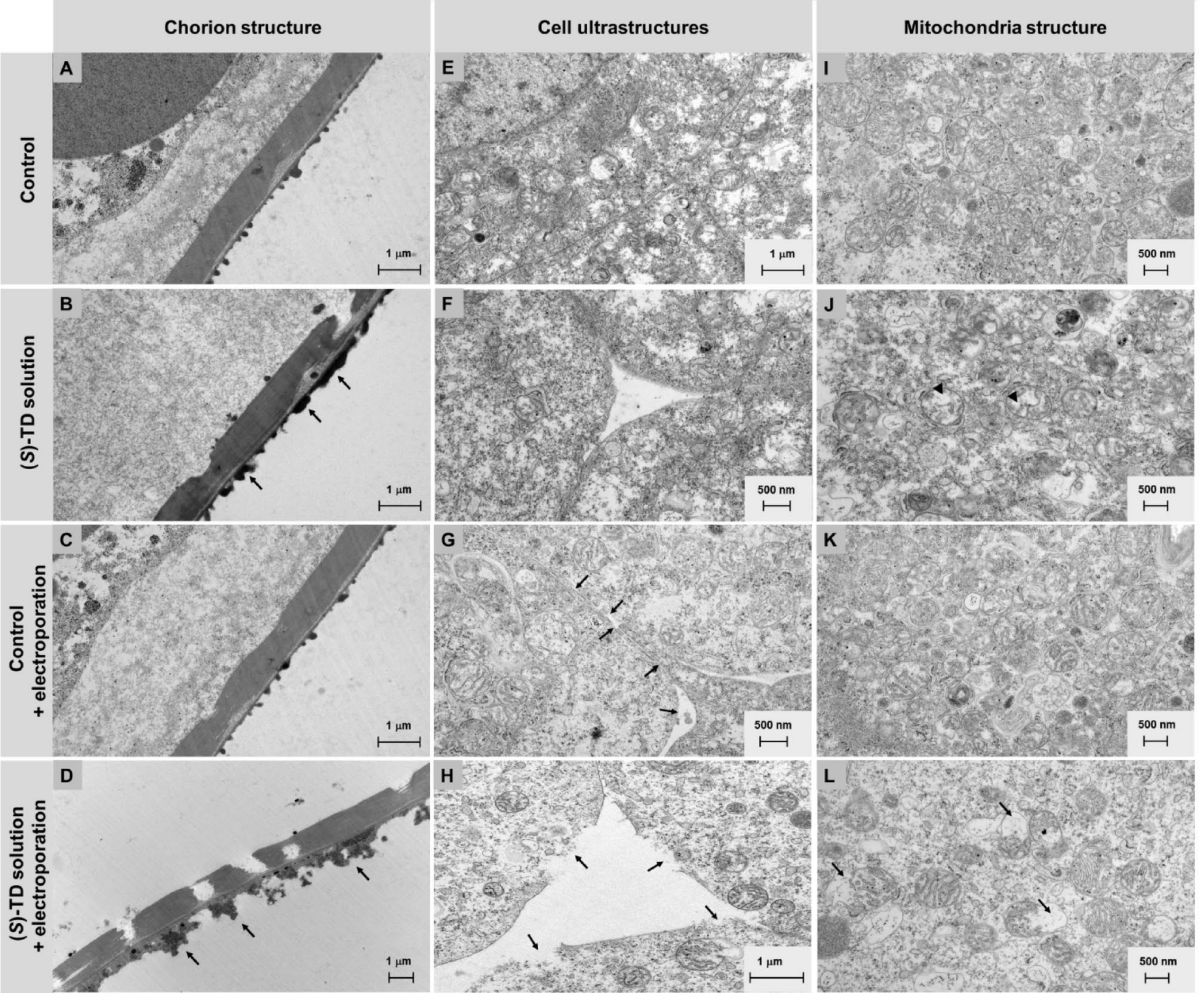
TEM images of chorion structure (**A**–**D**), cell ultrastructure (**E**–**H**), and mitochondria structure (**I**–**L**) of zebrafish embryos exposed to the (*S*)-thalidomide action and electroporation.

The effect of (*S*)-thalidomide on the structure of the chorion can be seen in the form of increased deposits of the outer layer (Fig. 4B and D, sites marked with arrows). In addition, the electroporation process led to the appearance of translucencies in the chorion structure and a marked widening of the layer immediately below the chorion (Fig. 4A and C). This is most likely due to the widening of intercellular spaces and the disruption of cellular connections. In turn, the analysis of the ultrastructure of the body-building cells of zebrafish embryos showed that under the influence of (*S*)-TD, there was a relaxation of the arrangement of cells forming the embryos. The distances between embryo cells were greatest after treatment of the embryo with (*S*)-thalidomide with pre-electroporation. In this variant, quite large cell membrane damage was also observed (indicated by arrows in Fig. 4H), greater than after electroporation alone (Fig. 4G). Cell loosening was also evident after treatment of embryos with (*S*)-TD alone (Fig. 4F), however, to a lesser extent. The control cells of the embryos (Fig. 4E), closely adhered to each other and the application of electroporation alone had little effect on the cell arrangement of the embryo, causing only the disruption of the cell membrane in the form of numerous pores marked by arrows in Fig. 4. These results are consistent with literature data^31^, which describe the effect of thalidomide on damaging intercellular junctions. In addition, there was also a change in the structure of mitochondria in the zebrafish embryo cells during the variants of the experiment carried out. The mitochondria of control cells were regular in shape, with numerous, regularly arranged ridges (Fig. 4I). The inner membrane of the mitochondria adhered to the outer membrane. The process of electroporation did not significantly affect the ultrastructure of the mitochondria of the embryonic cells (Fig. 4K). In contrast, changes in the ultrastructure were observed in the mitochondria of (*S*)-thalidomide treated embryonic cells. The arrangement of mitochondrial combs became irregular and there was a pronounced protrusion of mitochondria and their rupture (sites marked with arrows in Fig. 4L). The matrix of the altered mitochondria was clearly translucent (arrowheads in Fig. 4J). The most intense mitochondrial deformations were observed after thalidomide treatment with electroporation.

### Quantitative analysis of thalidomide enantiomers in Danio rerio embryos homogenates and solutions

**Fig. 5:**
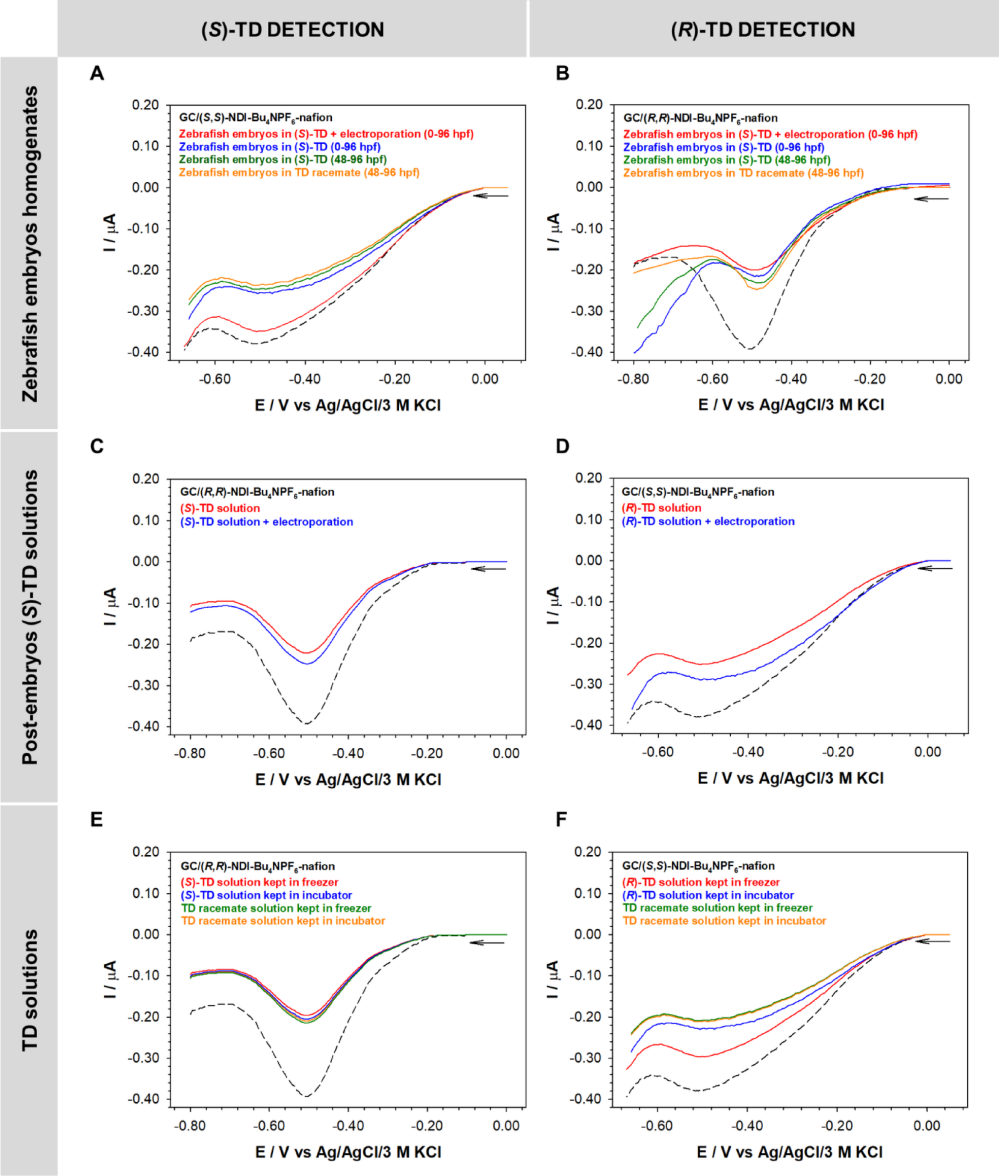
DPV voltammograms of NDI-receptors (GC/NDI-Bu_4_NPF_6_-nafion) recorded in zebrafish homogenates (**A**, **B**), solutions in which the embryos were kept (**C**, **D**), and pure TD solutions (**E**, **F**). Experimental conditions: mixture (1:1 v/v) of 1×PBS buffer (pH 7.4) and 0.02 M citrate buffer (pH 1.5) with an addition of 1% of DMSO.

Quantitative analysis of individual thalidomide enantiomers was carried out for Danio rerio embryos homogenates and solutions in which embryos were developed (E3 medium + 0.5% DMSO+ (*S*)-thalidomide) from 4 to 96 hpf (embryos with a chorion), additionally quantitative analyze was performed in embryos hatched at 48 hpf (exposure to thalidomide between 48 and 96 hpf). Zebrafish embryos (*n* = 20) at 96 hpf were homogenized in 200 µL of 0.5% DMSO before determination of thalidomide concentration. All samples were diluted 1000-fold in 1×PBS buffer before analysis. In order, to test the stability of the form of the TD enantiomer solutions of 0.8 mM (*S*)-thalidomide and the racemate stored under different conditions: the incubator (28 °C) and freezer (-20 °C) were additionally tested. The results of these studies were used to determine the effect of temperature on the stability and rate of transition of one form of thalidomide enantiomer into the other. The recorded DP voltammograms for both types of samples: embryos homogenates, pure (*S*)-TD solutions, and solutions in which zebrafish embryos were kept are shown in Fig. 5.

In all analyzed cases, the contact between the sensor and the solution resulted in a decrease in the current signal coming from the receptor (the corresponding chiral NDI derivative). The largest changes in current signal intensity were observed for (*S*)-thalidomide determinations regardless of the type of analyzed sample, as well as the method of its storage. In contrast, the changes in the current signal of the sensor sensitive to the presence of (*R*)-thalidomide were characterized by a statistically insignificant difference for zebrafish embryos exposed to electroporation. This indicates a high content of the (*S*)-thalidomide enantiomer in the samples and at the same time a low content of the (*R*)-thalidomide form. The results were slightly different for homogenates of embryos not exposed to electroporation at different stages of their development. Then the content of the (*R*)-TD enantiomer was equally significant. Higher concentrations of (*S*)-thalidomide were observed in embryos exposed to (*S*)-thalidomide and electroporated, while the opposite situation was observed in the solutions (post-embryos solutions) in which these embryos were immersed. Higher concentrations of (*S*)-thalidomide were in the wells in which the embryos were without exposure to electroporation. The determined concentrations of TD enantiomers in the analyzed solutions are given in Table 1. Thalidomide enantiomers are known to undergo natural interconversion in aqueous environments. The obtained results have shown that both the electroporation process and storage of (*S*)-TD solutions at low temperatures significantly reduce the transformation of one enantiomer into the other. Twice as high thalidomide concentrations were observed if exposure to thalidomide was given to embryos that were still in the chorion from 4 hpf (exposure 4–96 hpf) than to those that had already been exposed to thalidomide after leaving the chorion at 48 hpf (exposure (48–96 hpf). Brox et al. described higher concentrations of many substances for early exposure of zebrafish embryos with chorion compared to embryos dechorionated or exposed to compounds after leaving the chorion already at a later stage of development^32^.

**Table 1:** The content of individual TD enantiomers in: zebrafish homogenates, post-embryos (*S*)-TD solutions, and TD solutions kept in a freezer or incubator.

Zebrafish homogenates							
Zebrafish embryos in 0.8 mM (*S*)-TD solution (4–96 hpf)*		Zebrafish embryos in 0.8 mM (*S*)-TD solution + electroporation (4–96 hpf)*		Zebrafish embryos in 0.8 mM (*S*)-TD solution (48–96 hpf)**		Zebrafish embryos in 0.8 mM TD racemate solution (48–96 hpf)**	
(*S*)-TD [mM]	(*R*)-TD [mM]	(*S*)-TD [mM]	(*R*)-TD [mM]	(*S*)-TD [mM]	(*R*)-TD [mM]	(*S*)-TD [mM]	(*R*)-TD [mM]
0.394 ± 0.084	0.045 ± 0.013	0.686 ± 0.110	0.001 ± 0.002	0.176 ± 0.055	0.072 ± 0.022	0.100 ± 0.036	0.145 ± 0.029

Post-embryos (*S*)-TD solutions							
0.8 mM (*S*)-TD solution				0.8 mM (*S*)-TD solution + electroporation			
(*S*)-TD [mM]		(*R*)-TD [mM]		(*S*)-TD [mM]		(*R*)-TD [mM]	
0.256 ± 0.061		0.056 ± 0.010		0.094 ± 0.010		0.014 ± 0.006	

TD solutions							
0.8 mM (*S*)-TD solution kept in freezer		0.8 mM (*S*)-TD solution kept in incubator		0.8 mM TD racemate solution kept in freezer		0.8 mM TD racemate solution kept in incubator	
(*S*)-TD [mM]	(*R*)-TD [mM]	(*S*)-TD [mM]	(*R*)-TD [mM]	(*S*)-TD [mM]	(*R*)-TD [mM]	(*S*)-TD [mM]	(*R*)-TD [mM]
0.680 ± 0.021	0.010 ± 0.009	0.455 ± 0.060	0.150 ± 0.074	0.350 ± 0.036	0.345 ± 0.012	0.440 ± 0.056	0.300 ± 0.021

*Zebrafish embryos with chorion, **Zebrafish embryos without chorion.

### The potential of zero charge as an indicator of (*S*)-TD and membrane interaction

**Fig. 6:**
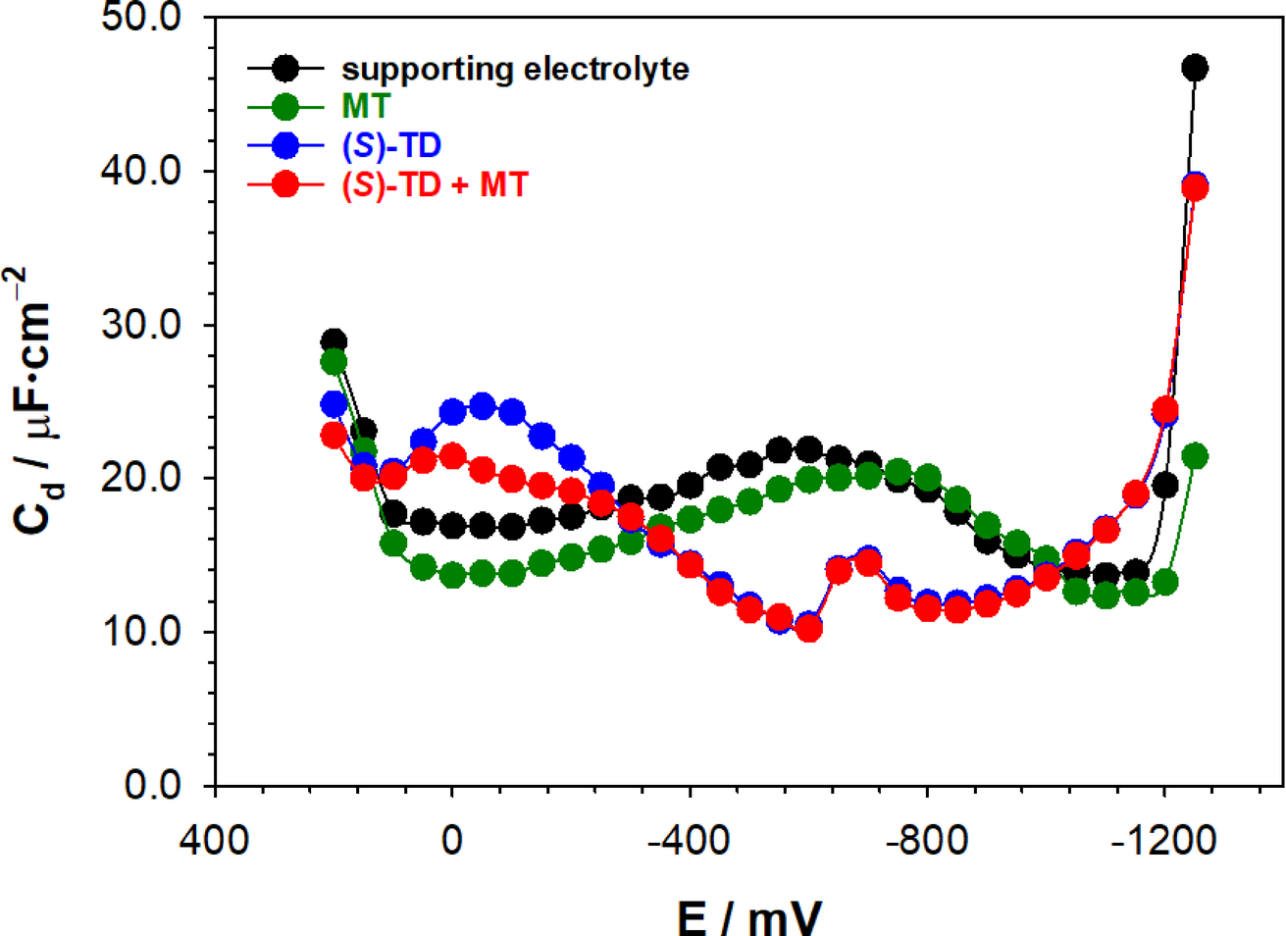
The differential capacity – potential curves of the double layer interface Hg in supporting electrolyte (2.0 mol·L^−1^ chlorate(VII)); 5.0·10^−4^ mol·L^−1^ MT; 5.0·10^−4^ mol·L^−1^ (*S*)-TD and 5.0·10^−4^ mol·L^−1^ (*S*)-TD + 5.0·10^−4^ mol·L^−1^ MT.

The surface charge and extensive adsorption properties of compounds determine the strength of the interaction between the selected compound and the cell membrane. To get information about this parameter the experiments using the method of CGMDE and streaming mercury electrode were performed. In the areas of the formation of the capacity “hump” (from about − 200 mV to − 1000 mV) after the introduction of L-methionine (MT) to the supporting electrolyte solution (see Fig. 6), its height decrease and moves significantly towards the negative potentials. Such a significant reduction of the differential capacity is associated with the adsorption properties of this amino acid.

The presence of (*S*)-TD in the supporting electrolyte solution caused a significant decrease in the differential capacity in the wide potential range (from − 200 mV to − 1000 mV), which indicates strong adsorption properties. In the region of higher potentials (≈ 0 mV) the adsorption peaks occur only in the presence of (*S*)-TD. It should be emphasized that on the differential capacity curves did not occur the (*S*)-TD desorption peak in the investigated potential range. The introduction of methionine into a solution containing (*S*)-TD does not drastically change the image of capacitive curves, besides the intensity decrease of the adsorption peak. This may indicate a change in the orientation of (*S*)-TD molecules on the electrode surface and co-adsorption of (*S*)-TD and MT molecules^33^. The addition of (*S*)-TD to the 2.0 mol·L^−1^ chlorates(VII) solution causes a shift of *E*_z_ towards more positive potentials (*E*_z_ = − 0.480 V for 2.0 mol·L^−1^ chlorates(VII) and *E*_z_ = − 0.468 V for (*S*)-TD), which indicates the adsorption of thalidomide molecules oriented with a positive pole to the mercury surface^34^. In turn, the introduction of specifically adsorbing methionine^34^ causes a clear shift of the *E*_z_ in the opposite direction (*E*_z_ = − 0.503 V for (*S*)-TD + MT), so towards more negative potentials. Such changes strongly indicate a change in the orientation of thalidomide molecules on the electrode surface and the co-adsorption of the TD and Mt molecules. Most likely, the TD molecules change their orientation from slanting to flat, thus allowing the aromatic ring π electrons to interact more easily with the electrode surface^35^. The values of surface tension *γ*_z_ at the zero charge potential decrease for all studied systems (*γ*_z_ = 478 mN·m^−1^ for 2.0 mol·L^−1^ chlorates(VII), *γ*_z_ = 475 mN·m^−1^ for MT; *γ*_z_ = 477.5 mN·m^−1^ for (*S*)-TD; *γ*_z_ = 477.2 mN·m^−1^ for (*S*)-TD + MT) as proved by the adsorption phenomenon.

## Conclusions

Zebrafish is an important vertebrate model representing an alternative approach in next-generation risk assessment. As a high throughput screening (HTS) model, it allows for a quick and, above all, a costly-accepted study of the potential toxicological effect with high predictive values. Considering a huge similarity of the zebrafish and human genomes^8^ the results obtained in this model constitute a valuable basis for studies of embryotoxic and teratogenic insults. The above allows for the estimation of many new toxicological endpoints with a high degree of probability, while maintaining a high accuracy of agreement in the prediction of interspecies extrapolation. Thalidomide is still the first-choice drug in such diseases as multiple myeloma and erythema nodosum leprosum, Crohn’s disease, Behcets disease, systemic lupus erythematosus and HIV infections. Therefore, monitoring the adverse effects in the embryonic period is of great importance in assessing the potential risk for pregnant women in clinical settings. The finding of spontaneous transformation of the (*R*)- and (*S*)-thalidomide enantiomers in the organism of zebrafish embryos is important evidence for the assessment of the risk into the human embryo. Zebrafish is currently an ideal model responding to physiologically relevant dose ranges and, when combined with cell-specific or tissue-specific effects in drug discoveries^36^. Our studies indicate that the use of discrete electrical pulses during electroporation can significantly facilitate the penetration of tested drugs through the chorion into the embryo, within the complexity of a whole embryo, transporting across embryonal tissues and over an extended timescale.

In this study the electroporation method was used to introduce (*S*)-thalidomide into the zebrafish embryos subjected to electroporation processes. Exposure of embryos to (*S*)-thalidomide under validated electroporation conditions produced massive body abnormalities such as shifts in body length and width, and deformations in the fins and changes of intracellular structures especially in the cell membrane and mitochondria. Such detrimental effects were also noted by other authors when thalidomide directly affected the zebrafish embryo devoid of the chorion by dechorionation^16,20,24^ or as a result of microinjection of thalidomide directly into the embryos^21^. In zebrafish embryos exposed to (*S*)-thalidomide and subjected to electroporation pulses, twice as high amount of (*S*)-thalidomide was determined compared to embryos not subjected to electroporation, which is a key evidence that the electroporation facilitates the penetration of (*S*)-thalidomide into the chorion interior. Our study showed the interconversion of thalidomide enantiomers in zebrafish embryos since it was possible to analyze the (*R*)-thalidomide after exposure to the pure (*S*)-thalidomide. Such effects have been also observed in humans. After intravenous administration of the (*S*)-enantiomer both the (*R*)- and (*S*)-thalidomide were detected in the blood^37,38^. In overall summary, our study indicates that electroporation is a promising method to increase the penetration of thalidomide through the chorion in the embryonal zebrafish model. The study performed on zebrafish embryos evidently confirms the teratogenic effects of (*S*)-thalidomide and its spontaneous conversions into the (*R*)-enantiomeric form in the embryos. Moreover, some novel mechanistic results of this drug were elucidated by studying the structure of the chorion on TEM and SEM images. This effect should be considered when using this drug in clinical situations. It seems that the results obtained in the zebrafish model may have important clinical implications. The possibility of a similar biotransformation in the human body indicates the need to monitor the stereo-enantiomers of this drug in clinical conditions. The above is justified not only in the assessment of therapeutic efficacy, but also in personalized control of adverse effects, which may result from the uncontrolled metabolism of the stereo-isoforms of this medicine. The above is of particular importance if the therapy process affects pregnant women and/or is directed to the human body in the pre- and early postnatal period. It should be emphasized that the study of embryotoxicity of new drug candidates using pregnant laboratory rodents is an ethically unacceptable on the basis of the new approach methods (NAMs) in toxicology, although it is still a regulatory permitted study. Therefore, studies using zebrafish embryos may represent a new methodological approach in this segment of preclinical safety assessment.

## Data Availability

Data for this article are available at https://danebadawcze.uw.edu.pl/dataset.xhtml? persistentId=doi:10.58132/CQNWBZ.
